# Comparative analysis of sex-based, vendor-based, and species differences in cytochrome P450 metabolism

**DOI:** 10.1038/s41598-025-34936-x

**Published:** 2026-01-13

**Authors:** Nivedita Kinatukara, Xin Xu, Pranav Shah

**Affiliations:** National Center for Advancing Translational Sciences (NCATS), 9800 Medical Center Drive, Rockville, MD 20850 USA

**Keywords:** CYP450, Sex differences, Vendor differences, Species differences, Biochemistry, Biological techniques

## Abstract

Hepatic clearance is crucial as it directly impacts drug exposure, efficacy, and safety. Cytochrome P450 (CYP450) enzymes play a pivotal role in drug metabolism and exhibit differences based on sex, species, and commercial liver microsome vendors. These variables can directly influence translational accuracy when preclinical data are applied to human drug development. In this study, we evaluated metabolic stability of isozyme-selective compounds across human, rat, and mouse liver microsomes, incorporating both male and female microsomes and multiple vendors. Our analysis revealed three layers of variability: (1) sex-specific differences consistent with prior clinical observations, where certain substrates displayed markedly faster clearance in one sex; (2) interspecies divergence, such as male-predominant isoforms in rodents without direct human orthologs; and (3) vendor-related discrepancies, where the same species-sex pool yielded divergent stability outcomes depending on microsome source. Together, these findings illustrate the combined effects of sex, species, and vendor source that contribute to variability in CYP450-mediated metabolism. By systematically comparing these factors, our work underscores the importance of considering these variables during early preclinical studies. Accounting for these sources of variability may improve the translational reliability of in vitro assays, reduce costly late-stage failures, and better support the development of safe and effective therapeutics.

## Introduction

Hepatic clearance is a key determinant of a compound’s systemic exposure, influencing dosing frequency, efficacy, and safety. In drug discovery, understanding hepatic clearance early helps optimize pharmacokinetic (PK) profiles and reduce late-stage development failures. Cytochrome P450s (CYP450s) are the most important drug-metabolizing enzymes in the liver involved in the metabolism of more than half of all clinically used drugs^1,2^. Given their significance, CYP450s have been extensively studied in clinical and drug development settings, with particular attention to sex-based differences in their activities^3,4^. Some of these earliest observations date back to 1932, when sex differences in barbiturate metabolism were described in rats^3^. Since then, numerous studies have documented sex differences in both the expression and activity of various CYP450 isozymes in humans, rats, and mice^1–5^. Notably, analyses of new drug applications reveal that 6–7% show at least a 40% difference in PK between sexes, underscoring the clinical impact of these findings^3^.

To build on this historical context, many of the studies exploring sex-based CYP variation rely on rodent models, which offer practical advantages because of their short lifespans and ease of breeding. While CYP450 families are broadly conserved at the sequence level, even minor structural differences can alter substrate specificity, inducibility, and its tissue distribution^1,5^. For example, rat Cyp2c11, the predominant male-specific isoform, has no direct human ortholog, whereas mouse Cyp2d22 is often considered the closest functional counterpart to human CYP2D6^6^. Similarly, rat Cyp3a2 is male-predominant but differs substantially in substrate selectivity and inducibility compared to human CYP3A4^5,7^. Rodents also typically exhibit faster drug metabolism due to higher hepatic CYP content relative to body weight^5,8^. Mouse and rat differences further complicate translation: male rats metabolize many compounds faster via male-specific CYP isoforms (e.g., Cyp2c11, Cyp3a2), while mice show milder sex dimorphism and, in some cases, female-predominant metabolism^9,10^. Additionally, for CYPs where clear rat orthologs are present, rat CYPs demonstrate high degrees of structural similarity to their human orthologs and, despite differences, provide valuable mechanistic insight into sex-specific regulation^1,5^. While general trends in sex-specific CYP activity are observed across species, significant differences in isozyme expression patterns and regulation remain. These interspecies differences emphasize limitations of using rodent models to predict human drug metabolism^5^ and the need for better understanding these differences.

The relevance of these limitations is highlighted by a real-world disconnect observed in our lab. During lead optimization, several compounds displayed favorable absorption, distribution, metabolism, and excretion (ADME) profiles. However, in PK studies, the lead candidate exhibited unexpectedly high clearance. Subsequent analyses revealed that the compound was metabolically unstable and produced oxidative metabolites not detected in earlier evaluations. Further investigation traced this discrepancy to the use of different microsome sources^11^. Subsequent studies in the disease model, which employed female mice, revealed markedly different PK, underscoring how both sex and vendor can influence translational predictions. This finding prompted us to investigate how often such discrepancies arise within a drug discovery setting.

In this context, this paper compiles evidence of sex-specific CYP450 activity across humans, rats, and mice by evaluating known compounds with high selectivity for individual CYP isozymes, analyzing their metabolic stability across species, sexes, and microsome vendors to assess both biological and source-related variability. By synthesizing literature and validating with experimental assays, we provide a framework for interpreting sex differences in drug metabolism and to better understand translational relevance.

## Materials and methods

### Compound selection for in vitro microsomal metabolic stability study

This work was motivated by a striking 12-fold difference observed in male versus female exposure in rats for one of NCATS’s drug discovery projects, emphasizing the need to consider sex as a variable in PK and metabolism studies. To systematically evaluate CYP450 activity across human, rat, and mouse microsomes, we assembled a panel of isoform-selective substrates from literature that represent the major drug-metabolizing CYPs (Table 1).

Some CYPs had only a handful of substrates exclusively metabolized by a single isoform, limiting data interpretation. To better capture trends, we added compounds that are predominantly metabolized by a given isoform and grouped them with their corresponding major isoforms. Substrates that showed contributions from multiple CYP isoforms with known secondary isoforms, were noted in the table to provide context for interpretation. All compounds were supplied by NCATS Compound Management to ensure standardized sourcing and handling.

**Table 1:** List of compounds used in this study based on selectivity per CYP450 isoform.

Substrate	Secondary Isoforms	Substrate	Secondary Isoforms	Substrate	Secondary Isoforms
**CYP3A4**		**CYP2D6**		**CYP2C9**	
Cyclosporine^4^	N/A	Venlafaxine^12^	N/A	S-warfarin^13^	N/A
Naloxegol^12^	N/A	Tolterodine^12^	N/A	Glimepiride^14^	N/A
Ivacaftor^15^	N/A	Desipramine^12^	N/A	Piroxicam^16^	N/A
Lurasidone^12^	N/A	Dextromethorphan^12^	N/A	Tolbutamide^17^	N/A
Testosterone^12^	N/A	Chlorpheniramine^18^	N/A	Meloxicam^19^	CYP3A4
Simvastatin^12^	N/A	Carvedilol^20^	CYP2C9	Torsemide^21^	CYP2C8
Terfenadine^22^	CYP2D6	Tetrabenazine^23^	CYP1A2	Losartan^24^	CYP3A4
Buspirone^12^	CYP2D6	Nortriptyline^25^	CYP2C19, CYP1A2	Diclofenac^26^	CYP3A4
Glibenclamide^27^	CYP2C9	Propranolol^28^	CYP1A2		
Ifosfamide^29^	CYP2B6				
Ondansetron^30^	CYP2D6, CYP1A2				
Flibanserin^31^	CYP2C19				
Bosentan^32^	CYP2C9				
**CYP2C19**		**CYP2C8**		**CYP1A2**	
S-mephenytoin^12^	N/A	Selexipag^12^	N/A	Duloxetine^12^	N/A
Omeprazole^12^	N/A	Amodiaquine^33^	N/A	Tasimelteon^12^	N/A
Citalopram^34^	CYP3A4	Tucatinib^35^	CYP3A4	Alosetron^12^	N/A
Diazepam^36^	CYP3A4	Enzalutamide^37^	CYP3A4	Tizanidine^12^	N/A
Esomeprazole^38^	CYP3A4	Paclitaxel^39^	CYP3A4	Caffeine^12^	N/A
Pantoprazole^40^	CYP3A4	Repaglinide^41^	CYP3A4	Phenacetin^42^	1A1
		Rosiglitazone^43^	CYP2C9	Olanzapine^44^	CYP2D6
		Dasabuvir^45^	CYP3A4	Theophylline^46^	CYP2E1
		Pioglitazone^47^	CYP3A4	Pirfenidone^48^	CYP2C19
**CYP2B6**				Clozapine^44^	CYP3A4; CYP2D6
**Substrate**	**Secondary Isoform**			Tacrine^49^	CYP3A4; CYP2D6
Propofol^50^	N/A				
Efavirenz^51^	N/A				
Artemisinin^27^	CYP3A4				
Bupropion^12^	*several CYP isoforms				

N/A = Not Applicable.

### Microsomal stability assay

The substrate depletion method was used to determine compound half-life (t₁/₂) by tracking parent compound disappearance over time. Incubations were run using a Tecan EVO 200 system with a 96-channel head and Inheco heating/cooling block. Sources of microsomal fractions are listed in Table 2. Vendors were chosen based on their availability of sex-specific pooled microsomes and their broad use in preclinical ADME research. All microsomal fractions were vendor-supplied pooled lots, with one pooled lot used for each condition. Assay controls, internal standard (albendazole), and buffers were obtained from Sigma-Aldrich (St. Louis, MO). Reactions (110 µL) contained 1 µM test compound (prepared from 10 mM DMSO stocks diluted in 1:2 acetonitrile: water; final organic concentration i.e. DMSO + acetonitrile < 1%), 0.5 mg/mL microsomal fraction, and NADPH system (Corning-Gentest; Woburn, MA) in phosphate buffer (pH 7.4), incubated at 37 °C and sampled at 0, 5, 10, 15, 30, and 60 min. At each timepoint, 10 µL was quenched with cold acetonitrile/internal standard, centrifuged (3000 rpm, 20 min, 4 °C), and supernatants were transferred to analysis plates. Quantification was done via Thermo UHPLC/HRMS and processed using TraceFinder 4.1 and Validator software as described previously^52,53^. For each isoform, all sex, species, and vendor comparisons are based on the same compound sets tested in parallel. Since Corning-Gentest did not carry human female pooled liver microsomes, human datasets were only derived from Xenotech microsomal fractions.

**Table 2:** Source catalog numbers and lot numbers for microsomal fractions used in the study.

		Male		Female	
		Catalog #	Lot #	Catalog #	Lot #
Corning-Gentest (now Discovery Life Sciences)	Mouse Liver Microsomes	452701	2307301	452702	2402021
	Rat Liver Microsomes	452501	2303215	452502	2503131
Xenotech (now BioIVT)	Human Liver Microsomes	MX00801	2310283	FX00801	2310343
	Mouse Liver Microsomes	M1000	2410002	F00501	JRE
	Rat Liver Microsomes	R1000	1410271	F000100	1110040

### Data analysis

Relative microsomal stability that compares the difference between variables (i.e. sex or vendor) was defined as the log₁₀-transformed fold change in half-life values between two groups, scaled by the magnitude of their difference, allowing consistent comparison across sex, vendor, and species. The following equation was used in Excel, where A1 is the baseline group and A2 is the comparison group: =IF(A2 > A1, (A2 – A1) * LOG(A2 / A1, 10), -(A1 – A2) * LOG(A1 / A2, 10)).

## Results and discussion

### CYP3A4

CYP3A4, responsible for metabolizing ~ 50% of marketed drugs, exhibits well-documented sex differences in humans, with higher activity in females linked to faster drug clearance^3,54,55^. This female predominance has been attributed to estrogen-mediated activation of nuclear receptors, such as pregnane X receptor (PXR) and constitutive androstane receptor (CAR), and to continuous growth hormone (GH) secretion patterns in women^7,56^. Clinically, higher CYP3A4 activity in women is associated with faster clearance of drugs such as midazolam, verapamil, and cyclosporine, and increased adverse drug reactions have been reported in female patients^57^.

**Fig. 1:**
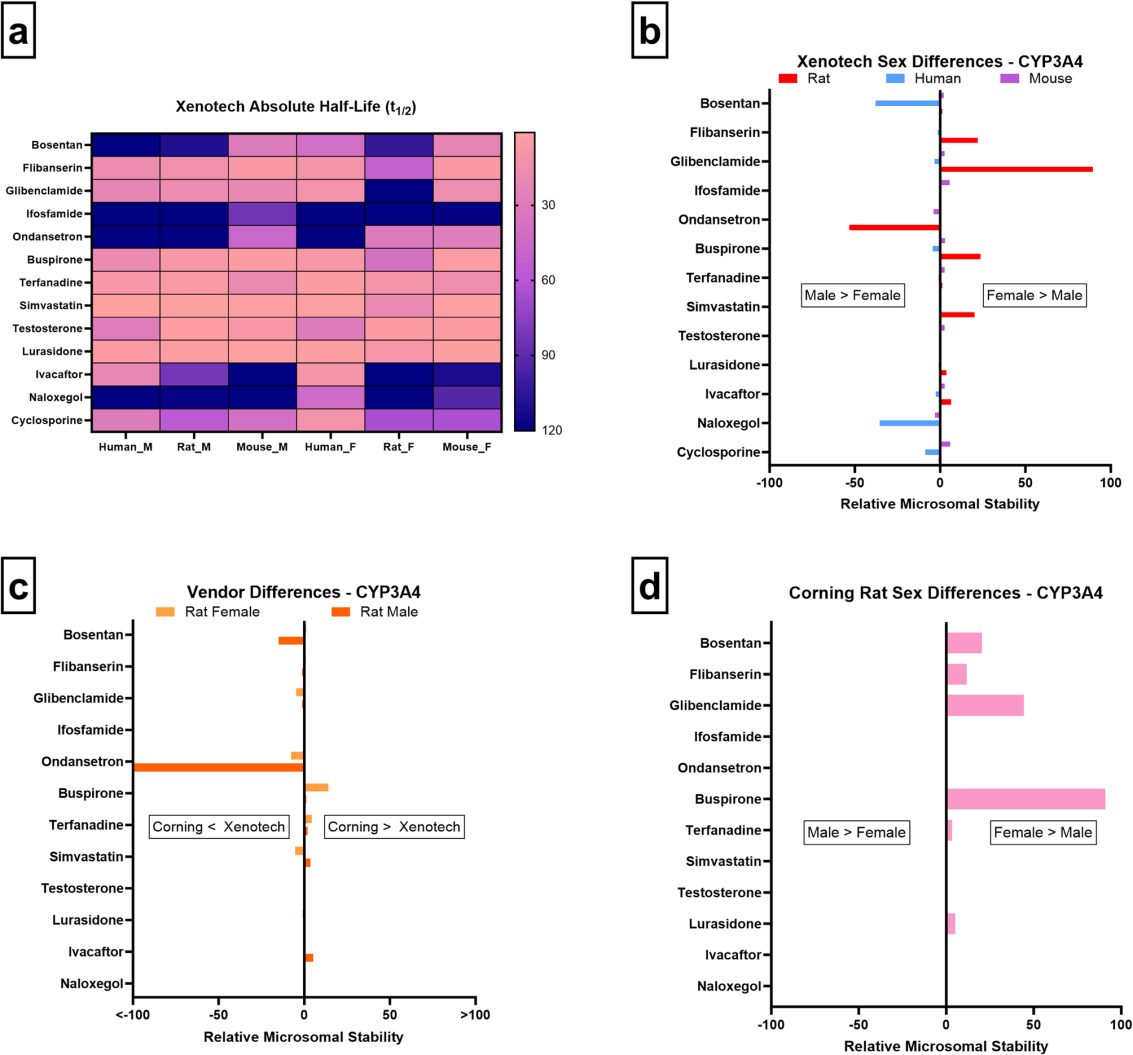
CYP3A4 microsomal stability comparisons across sex, vendor, species, and absolute half-life values. (**a**) Heatmap of absolute half-life values (minutes) for each substrate across male and female human, rat, and mouse Xenotech microsomes. Color scale indicates relative half-life magnitude (lighter = shorter half-life, darker = longer half-life). (**b**) Sex-based differences in microsomal stability in different species: log₁₀-transformed fold change magnitudes for Xenotech female relative to Xenotech male across human (blue), rat (red), and mouse (purple). (**c**) Vendor-based differences in rat microsomal stability: log₁₀-transformed fold change magnitudes for Corning rat microsomes relative to Xenotech comparing the same sex with each other; females (light orange) and males (dark orange). (**d**) Sex-based differences in microsomal stability of Corning rats: log₁₀-transformed fold change magnitudes for female relative to male (baseline).

Our data are consistent with known trends in humans: bosentan, naloxegol, and cyclosporine are more stable in male microsomes, indicating higher CYP3A4 activity in females (Fig. 1a/b). Conversely, in rats, flibanserin, glibenclamide, buspirone, and simvastatin show lower stability in males. No major sex differences are seen in mice.

Vendor comparisons in rats reveal no consistent patterns (Fig. 1c), suggesting that sex rather than vendor effects primarily drive these differences. When assessing sex differences using Corning rat and mouse microsomes, the results show consistent sex-related trends in rats, with female microsomes displaying lower activity than male microsomes for most compounds (Fig. 1d), whereas no such differences are observed in mice.

Overall, these results support existing literature that CYP3A4 activity is female-predominant in humans while activity of the orthologous rodent isoform, Cyp3a2, is male-predominant in rats, driven by rodent-specific GH-dependent regulation^3,27^. This highlights the translational challenge of interspecies differences in CYP regulation and underscores the importance of interpreting preclinical data within the context of human-specific hormonal and receptor-mediated control of CYP3A4 activity.

### CYP2D6

CYP2D6 is responsible for metabolizing ~ 30% of clinically used drugs, including antidepressants, β-blockers, opioids, and antitussives, despite comprising only ~ 2–5% of hepatic CYP content^5,58^. Unlike CYP3A4, sex differences in CYP2D6 activity are not as well established and are often overshadowed by genetic polymorphisms that define poor, intermediate, extensive, and ultra-rapid metabolizer phenotypes^2,4^. These polymorphisms significantly influence therapeutic response, particularly for opioids such as codeine, where ultra-rapid metabolizers experience heightened analgesia and adverse effects, while poor metabolizers see limited efficacy^59^.

**Fig. 2:**
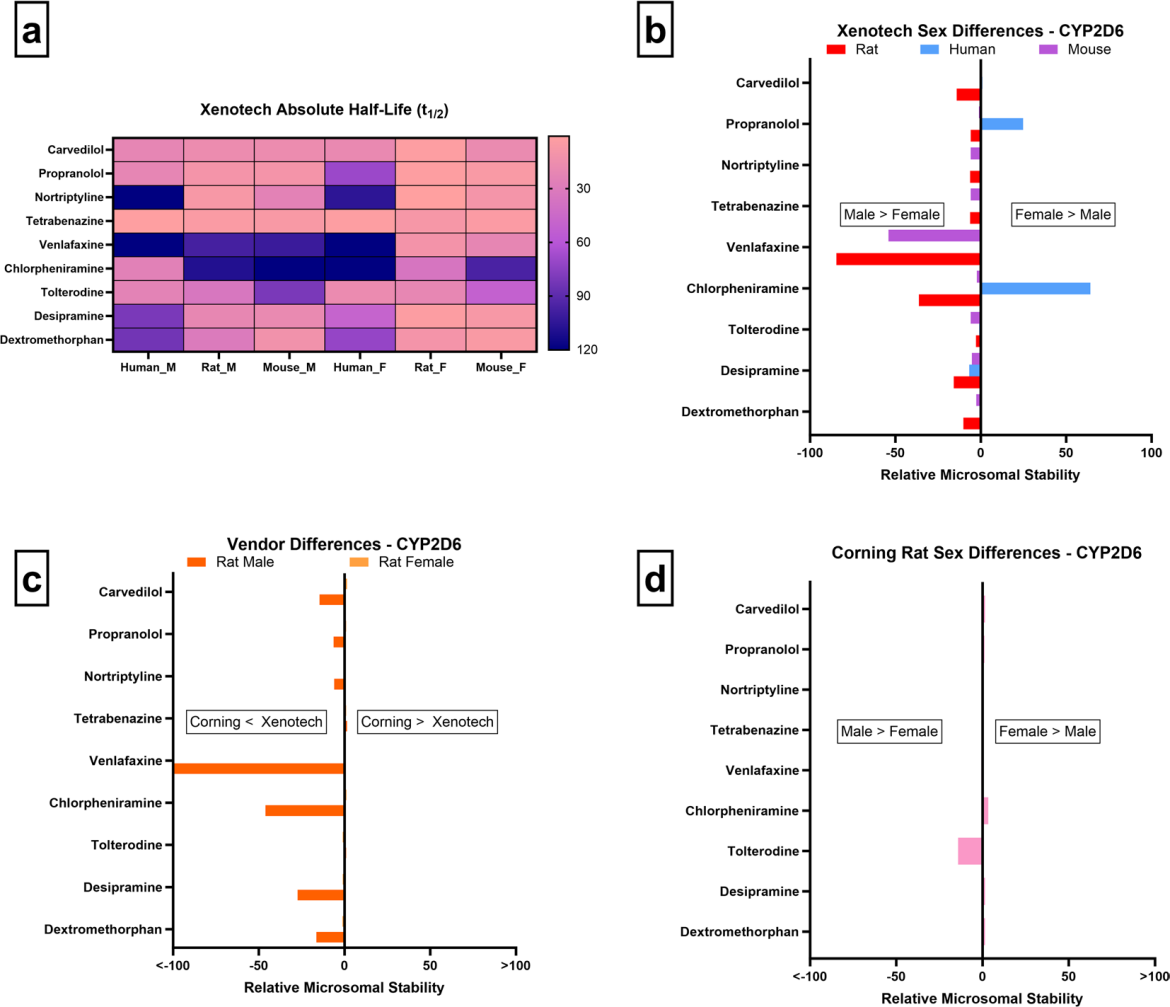
CYP2D6 microsomal stability comparisons across sex, vendor, species, and absolute half-life values. (**a**–**d** as above)

While no trends are observed in human or mouse, notable sex differences are observed in rat: compounds such as carvedilol, propranolol, nortriptyline, tetrabenazine, venlafaxine, chlorpheniramine, desipramine, and dextromethorphan exhibit lower stability in females, suggesting higher rat Cyp2d-mediated activity (Fig. 2a/b). However, these differences were not evident in Corning derived fractions, where male and female microsomes show comparable stability (Fig. 2d). Vendor comparisons provide further context: Xenotech males display consistently higher stability than Corning males across the same compounds (Fig. 2c), mirroring the sex-related differences within the Xenotech set. This pattern suggests that the attenuated activity in Xenotech males may amplify apparent sex differences within that vendor, potentially reflecting differences in colony genetics or baseline enzyme expression. Our lab has previously observed vendor-driven differences in rat Cyp2d activity^11^, supporting this interpretation. We also assessed sex differences using Corning mouse microsomes, however no differences were found, and the data is consistent with Xenotech mouse microsome data in Fig. 2b.

These findings partially diverge from prior literature describing rat Cyp2d1 (the ortholog of human CYP2D6) as male-predominant under GH-regulated conditions^6,9^. Instead, our results suggest that vendor-related variability in enzyme activity may obscure or alter expected sex-driven patterns in rats, emphasizing the need to consider both sex and supplier when interpreting Cyp2d data. A very interesting study is the recent case of E2027, a lead compound in development for treating dementia, displayed marked PK variability in Sprague-Dawley rats, with animals segregating into high- and low-clearance groups. Plasma AUC in the low-clearance group was ~ 11-fold higher than in the high-clearance group after oral dosing. Mechanistic investigations revealed that E2027 is primarily metabolized by rat Cyp2d1 and Cyp3a1, and that rat Cyp2d1 polymorphism drives the observed inter-individual PK variability of E2027^60^. Given the prevalence of such polymorphisms in rats and our in vitro findings, it is plausible that the rat colony used to produce Xenotech microsomes harbors spontaneous Cyp2d polymorphisms. Another potentially interesting point is that these polymorphisms might be exclusive to the male colony, as no vendor differences were noted in the stability results from female microsomes across the two vendors.

In humans, CYP2D6 activity is shaped primarily by individual polymorphisms rather than sex^2,4^, with poor, intermediate, extensive, and ultra-rapid metabolizer phenotypes accounting for most interindividual variability. Clinically, this is exemplified by opioids: ultra-rapid metabolizers experience both greater analgesia and higher adverse event risk, while poor metabolizers derive minimal efficacy^59^. Notably, this same clinical study also reported that women across normal-to-intermediate metabolizer phenotypes exhibited significantly more adverse effects, suggesting potential interactions between sex hormones and CYP2D6 phenotype even in the absence of large baseline sex differences in enzyme activity^59^.

Collectively, these findings demonstrate that while sex differences in CYP2D6-related activity are evident within Xenotech microsomes, vendor-driven variability in enzyme activity likely amplified these effects. This vendor influence underscores the need for careful interpretation of preclinical data, as rat Cyp2d sex differences may not only be exaggerated relative to humans but also shaped by supplier-specific colony factors. Given that human CYP2D6 variability is dominated by polymorphisms and clinical evidence links CYP2D6 phenotype to sex-dependent adverse events^59^, integrating both sex- and vendor-controlled assessments is critical to improve the translational value of early CYP2D6 metabolism studies.

### CYP2C9

CYP2C9 plays an essential role in metabolizing widely used drugs, including anticoagulants, non-steroidal anti-inflammatory drugs, and sulfonylureas. It has been reported that even modest decreases in activity can raise the risk of adverse events such as warfarin-associated bleeding or glimepiride-induced hypoglycemia^54,57^.

**Fig. 3:**
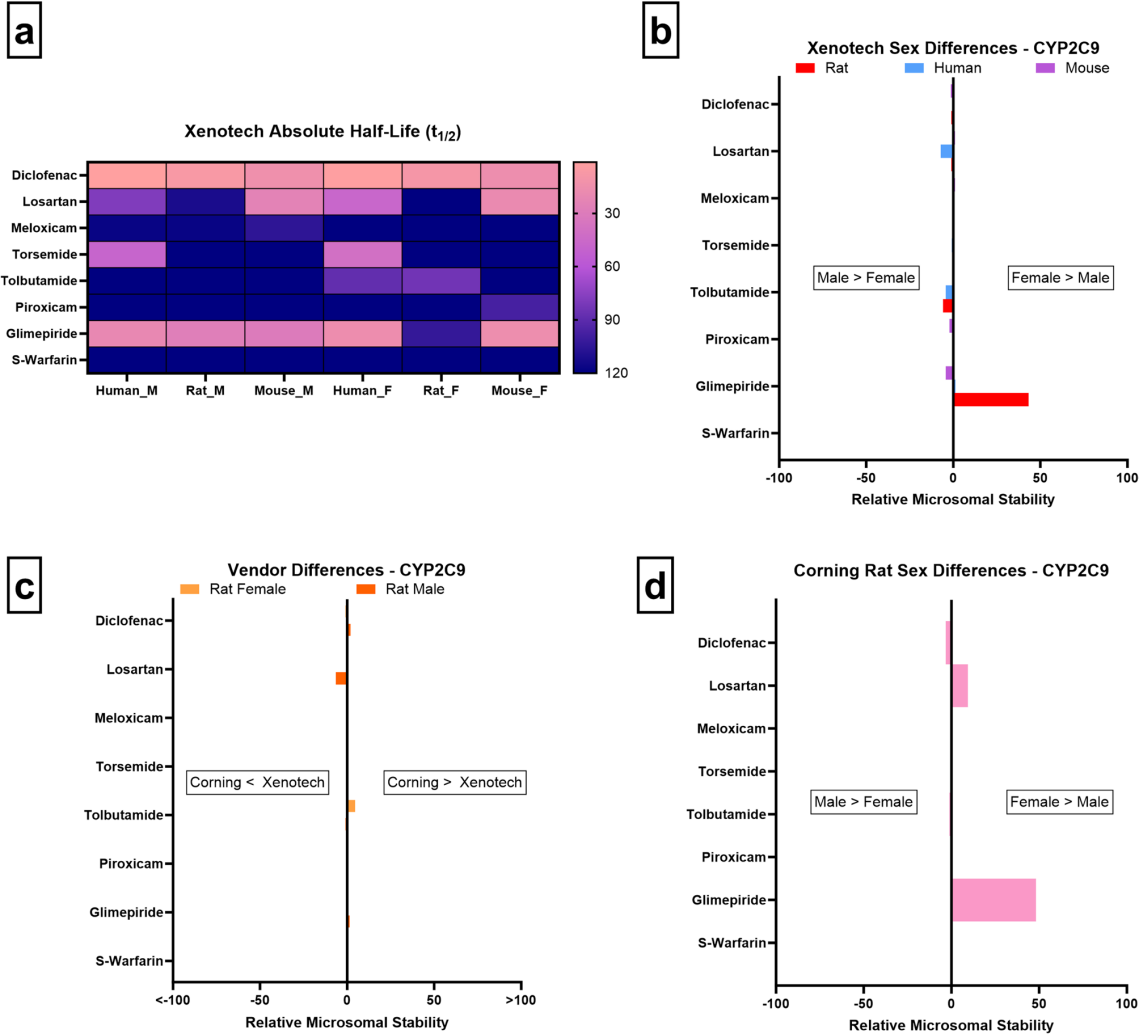
CYP2C9 microsomal stability comparisons across sex, vendor, species, and absolute half-life values. (**a**–**d** as above)

In our study, glimepiride demonstrates greater stability in female rats than males, with no notable vendor-related differences. No clear sex-related trends emerge in mice or humans (Fig. 3a/b/c). Glimepiride stability differences in Corning rats parallel the primary findings (Fig. 3d) whereas no significant sex-related stability differences are observed in Corning mice.

Human studies from literature largely indicate that sex is not a major determinant of CYP2C9 activity. One study reported no sex differences in CYP2C9 activity across 100 human liver samples despite pronounced interindividual variation^58^, while two others similarly found no clear sex effect in clinical PK data^1,4,55^. Instead, genetic polymorphisms and drug–drug interactions are implicated in variability^1,57^. In contrast, rodent studies demonstrate more pronounced sex-specific patterns tied to GH regulation. Male-predominant GH pulsatility induces higher hepatic expression of Cyp2c11, the major male-specific Cyp2c isoform in rats (without a direct ortholog to CYP2C9), while continuous GH secretion in females suppresses this expression^1,10,27,61^. This hormonal regulation drives faster drug clearance in male rats, while female rats express higher levels of Cyp2c12, a female-predominant rat isoform absent in humans^1,3,7^. Our observed female-predominant glimepiride stability in rats likely reflects this sex-dimorphic rodent Cyp2c profile rather than a translatable human trend.

Previous work has further highlighted the limitations of extrapolating rodent Cyp2c findings to humans. Martignoni^1,6^ emphasized that rats express multiple sex-regulated Cyp2c isoforms (Cyp2c7, Cyp2c11, Cyp2c12, Cyp2c13), none of which directly correspond to human CYP2C9. Meanwhile another study similarly noted that GH-driven sex dimorphism is exaggerated in rats compared to humans^1,8,9^, complicating translational relevance. Our findings, showing a lack of consistent sex effects in humans and mice despite rat-specific differences, align with these reports and reinforce that rodent-specific regulation must be contextualized when interpreting CYP2C9-mediated metabolism.

### CYP2C19

CYP2C19 is clinically relevant for the metabolism of proton pump inhibitors, selective serotonin reuptake inhibitors, and prodrugs such as clopidogrel. Genetic polymorphisms drive extensive interindividual variability, producing poor, intermediate, extensive, and ultra-rapid metabolizer phenotypes^54^. These polymorphic effects frequently outweigh sex differences in clinical contexts. Poor metabolizers exhibit impaired clopidogrel activation, increasing cardiovascular risk, while ultra-rapid metabolizers clear proton pump inhibitors more rapidly, reducing therapeutic efficacy. Current literature highlights mixed findings for sex differences in CYP2C19. One study reported modest female-biased CYP2C19 expression in human liver^55^, while another noted substrate-dependent trends^54^: S-mephenytoin clearance was faster in men, but other studies found no sex difference or even female-predominant activity. These discrepancies may reflect hormonal influences. Estrogen-containing oral contraceptives inhibit CYP2C19, as a clinical study showed with increased omeprazole exposure in women^62^, while pregnancy also reduces CYP2C19 activity^54,57^.

**Fig. 4:**
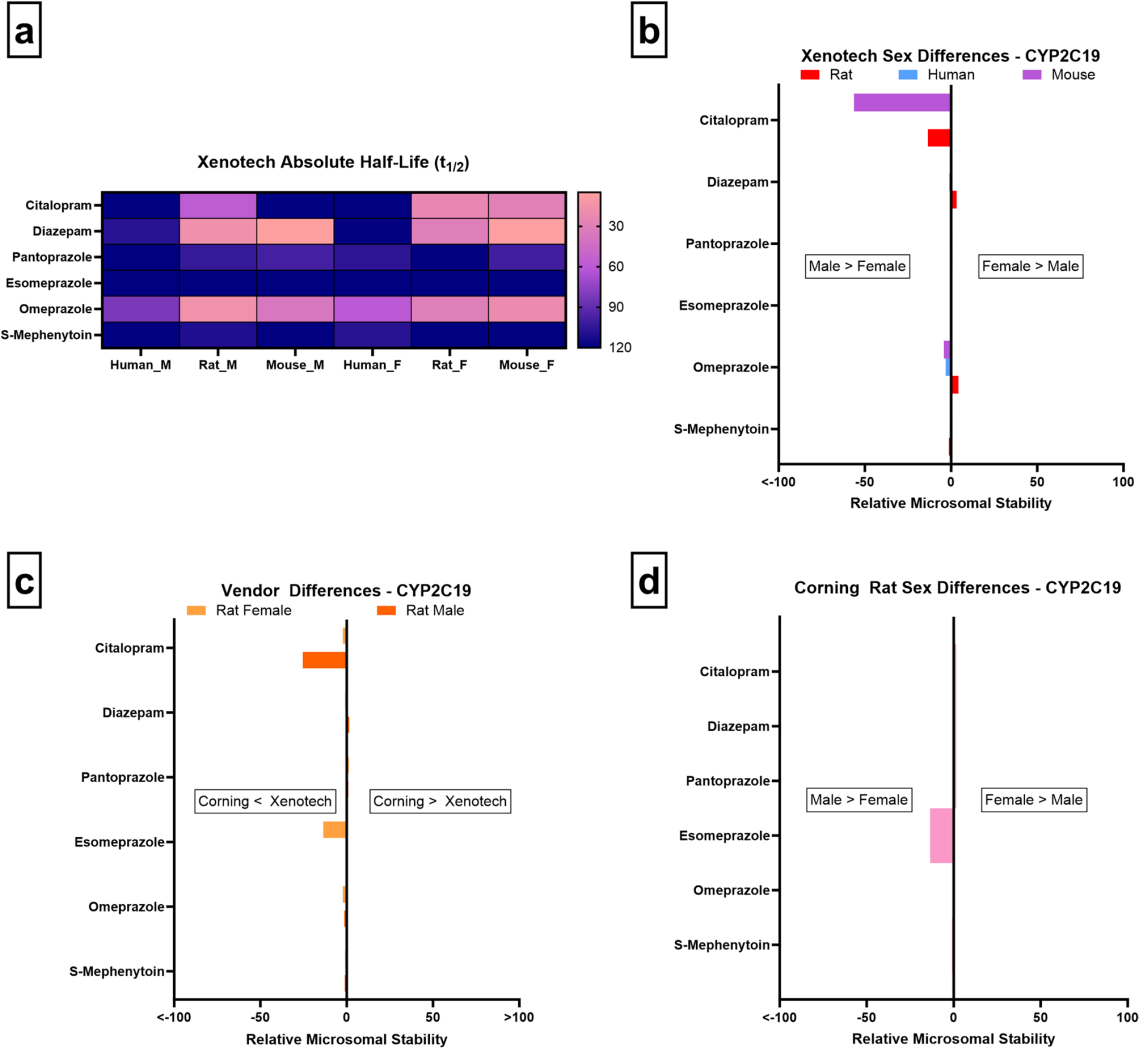
CYP2C19 microsomal stability comparisons across sex, vendor, species, and absolute half-life values. (**a**–**d** as above)

Of the six CYP2C19 substrates assessed in this study, only citalopram exhibited notable sex differences, showing greater stability in male rats and mice and suggesting lower enzymatic activity in these groups, while no other consistent sex-dependent patterns were observed across species (Fig. 4a/b). Vendor-related differences in rats (Fig. 4c) and sex-related differences in Corning rats (Fig. 4d) and mice (data not shown) were minimal. This indicates that hormonal status and genetic polymorphisms may play a larger role than sex alone in CYP2C19-mediated metabolism. In rodents, Cyp2c isoforms (e.g., Cyp2c29 in mice, Cyp2c6/7 in rats) metabolize CYP2C19 substrates but do not represent strict orthologs of human CYP2C19, contributing to the inconsistent sex-related patterns observed across species^5^. These results align with previous reports that CYP2C19 sex differences are modest compared to other CYPs (e.g., CYP3A4) and are often secondary to genotype or hormonal status.

### CYP2C8

CYP2C8, while less studied than other major isozymes, is responsible for metabolizing a diverse array of therapeutic agents, including chemotherapeutics, antidiabetics, and analgesics^5,8^.

**Fig. 5:**
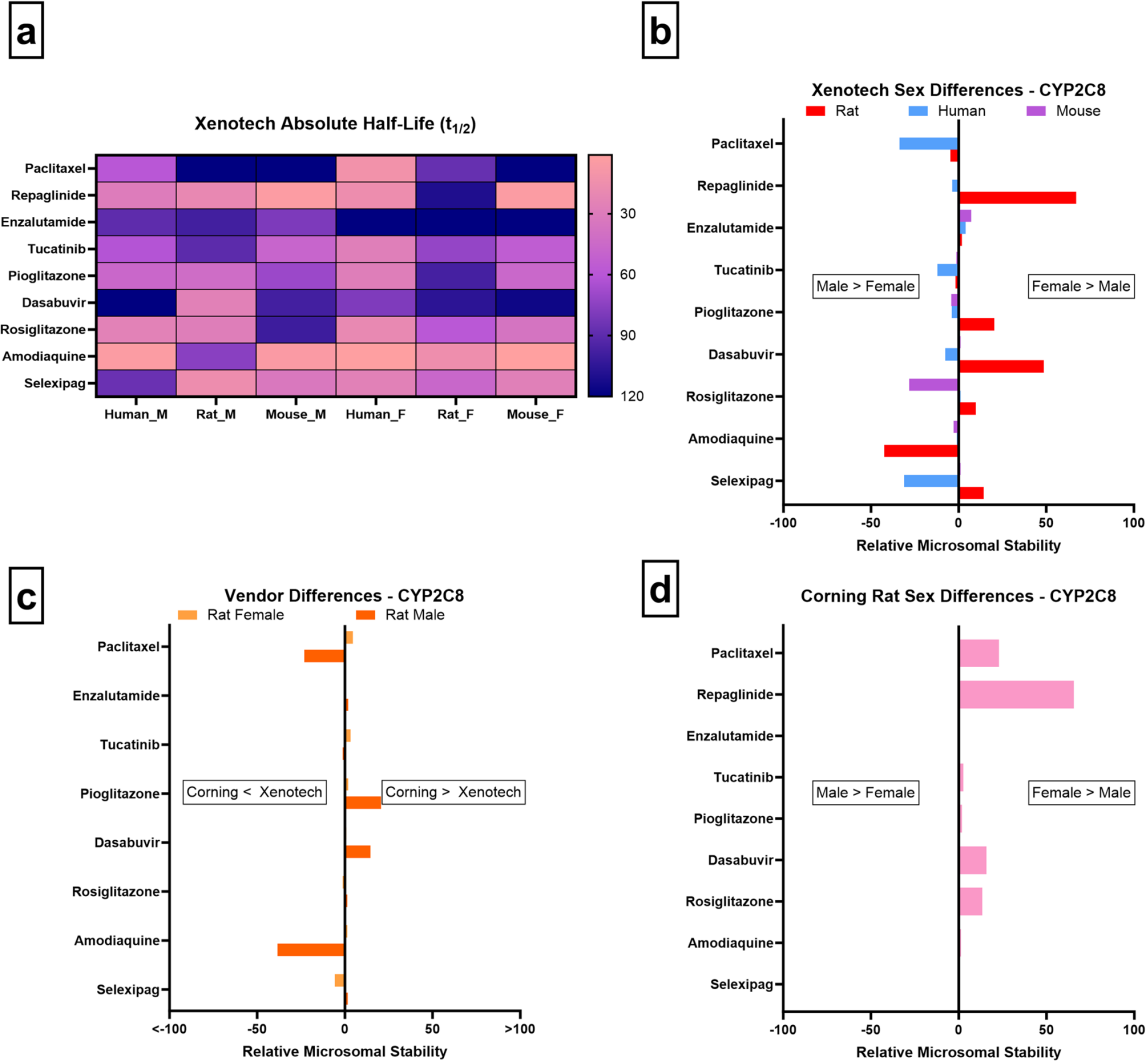
CYP2C8 microsomal stability comparisons across sex, vendor, species, and absolute half-life values. (**a**–**d** as above)

From our microsomal studies in rats, repaglinide, pioglitazone, dasabuvir, rosiglitazone, and selexipag are more stable in females, indicating lower enzyme activity, while amodiaquine shows the opposite trend. In humans, paclitaxel, tucatinib, dasabuvir, and selexipag are more stable in males, corresponding to higher CYP2C8 activity in women. Mouse data reveals minimal sex-related variation (Fig. 5a/b). Vendor comparisons in rats show four substrates with differences aligning with sex-based trends. Xenotech male rats metabolize dasabuvir and pioglitazone faster than Corning males, which mirrors the higher stability seen in Xenotech females. Similarly, amodiaquine displays higher stability in Xenotech males compared to both Xenotech females and Corning males (Fig. 5c). Notably, Corning male and female rats show similar amodiaquine stability, underscoring vendor-specific variability that may incorrectly highlight sex differences (Fig. 5d). We also assessed sex differences using Corning mouse microsomes but found no differences, consistent with Xenotech mouse data (Fig. 5b). It is important to note that rodents lack a true ortholog of human CYP2C8. Instead, metabolism of CYP2C8 substrates in rats and mice is mediated by multiple Cyp2c isoforms with overlapping but not identical substrate specificities.

These findings align with known sex-related CYP2C8 variability in both humans and rodents. In humans, higher CYP2C8 activity in females has been linked to faster clearance of substrates such as paclitaxel and selexipag^8,55,57^, a trend supported by our human data. This female predominance has been attributed to hormonal influences, including estrogen-mediated modulation of nuclear receptors, and sex differences in GH secretion patterns that indirectly regulate P450 expression^7,8,27^. Conversely, rodent studies show male-predominant Cyp2c activity, driven by pulsatile GH secretion in males and continuous GH secretion in females, leading to divergent sex effects between species^3,8,10^.

Collectively, our results corroborate that CYP2C8 activity is likely sex-dependent and species-specific, consistent with known hormonal and transcriptional regulation of rodent and human P450s^3,8,55^. Moreover, the vendor-specific differences observed highlight how early-stage preclinical studies must integrate both sex and microsomal sources to better predict human-relevant drug metabolism. Failure to account for these variables may mask important trends, particularly for isozymes like CYP2C8 that already exhibit moderate sex differences and substantial inter-individual variability.

### CYP2B6

CYP2B6, though historically considered a minor isozyme, now accounts for the metabolism of approximately 25% of marketed drugs and exhibits significant inter-individual variability^5,8^. Human studies have reported higher CYP2B6 expression and activity in women, influenced by estrogen-responsive transcriptional mechanisms^2,8,57^, although these differences are modest compared to other CYPs. Moreover, pregnancy and oral contraceptive use have been associated with enhanced CYP2B6 activity^8,57^, reflecting hormonal regulation. In rodents, however, Cyp2b expression is typically male-predominant, driven by pulsatile GH secretion and suppressed in females by continuous GH release^3,8,10^. This divergence underscores species-specific regulation of Cyp2b isoforms, with rat orthologs (Cyp2b1/2) displaying distinct sex-based differences in expression patterns compared to human CYP2B6^5,8,27^.

**Fig. 6:**
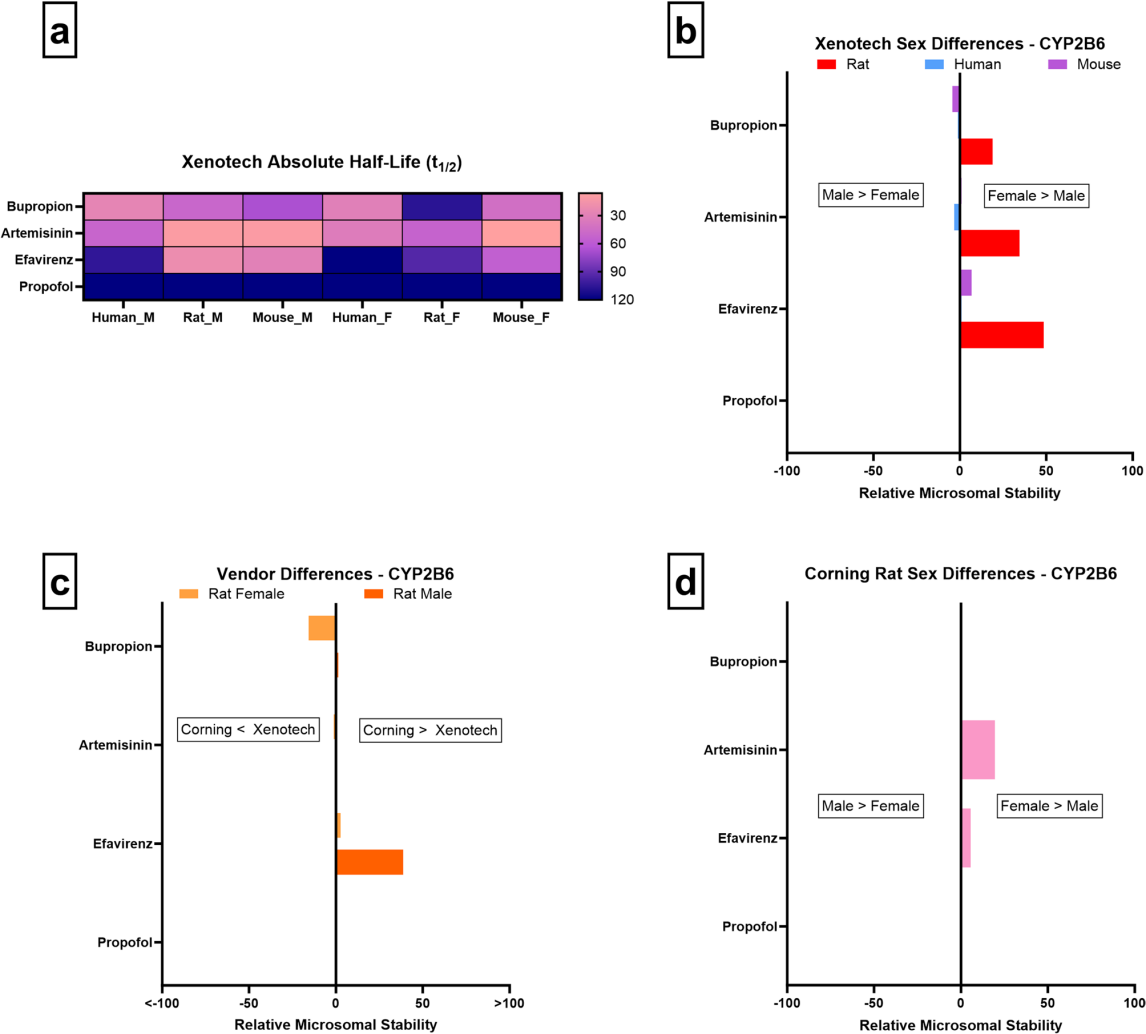
CYP2B6 microsomal stability comparisons across sex, vendor, species, and absolute half-life values. (**a**–**d** as above)

In our study, with a limited compound pool of 4 substrates, bupropion, artemisinin, and efavirenz are more stable in females than male rats, indicating reduced activity in females (Fig. 6a/b). No consistent vendor-driven differences emerge when testing the same compounds across vendors, aside from efavirenz, which displays slightly higher stability in Xenotech males than Corning males (Fig. 6c). Human and mouse data show minimal sex-dependent variation (Fig. 6a/b), aligning with prior findings that hormonal influences on CYP2B6 are modest and that genetic polymorphisms (e.g., CYP2B6*6) exert greater impact on the metabolic activity than sex alone^2,55^. Sex differences in Corning mouse microsomes were evaluated, but none are observed, aligning with Xenotech mouse data (Fig. 6b).

Taken together, our results support prior evidence that CYP2B6 demonstrates relatively limited sex differences compared to other CYP enzymes such as CYP3A4. The male-predominant activity in rats versus sex-neutral findings in humans illustrates the translational constraints of rodent models for this isozyme. Additional studies may be needed to fully understand this CYP isoform.

### CYP1A2

CYP1A2 is a key hepatic enzyme responsible for metabolizing roughly 13% of clinically used drugs and is known for male-predominant activity in humans^1–4,63^. Higher CYP1A2 clearance rates in men have been documented using probe substrates such as caffeine and antipsychotics, resulting in lower plasma concentrations in men compared to women^1,54,57^. Notably, olanzapine and clozapine, both metabolized primarily by CYP1A2, exhibit sex-related differences in therapeutic response and adverse effects, with women showing improved efficacy but increased side effects^3^. Induction factors such as smoking further complicate CYP1A2 activity, given its strong inducibility^2^ .

**Fig. 7:**
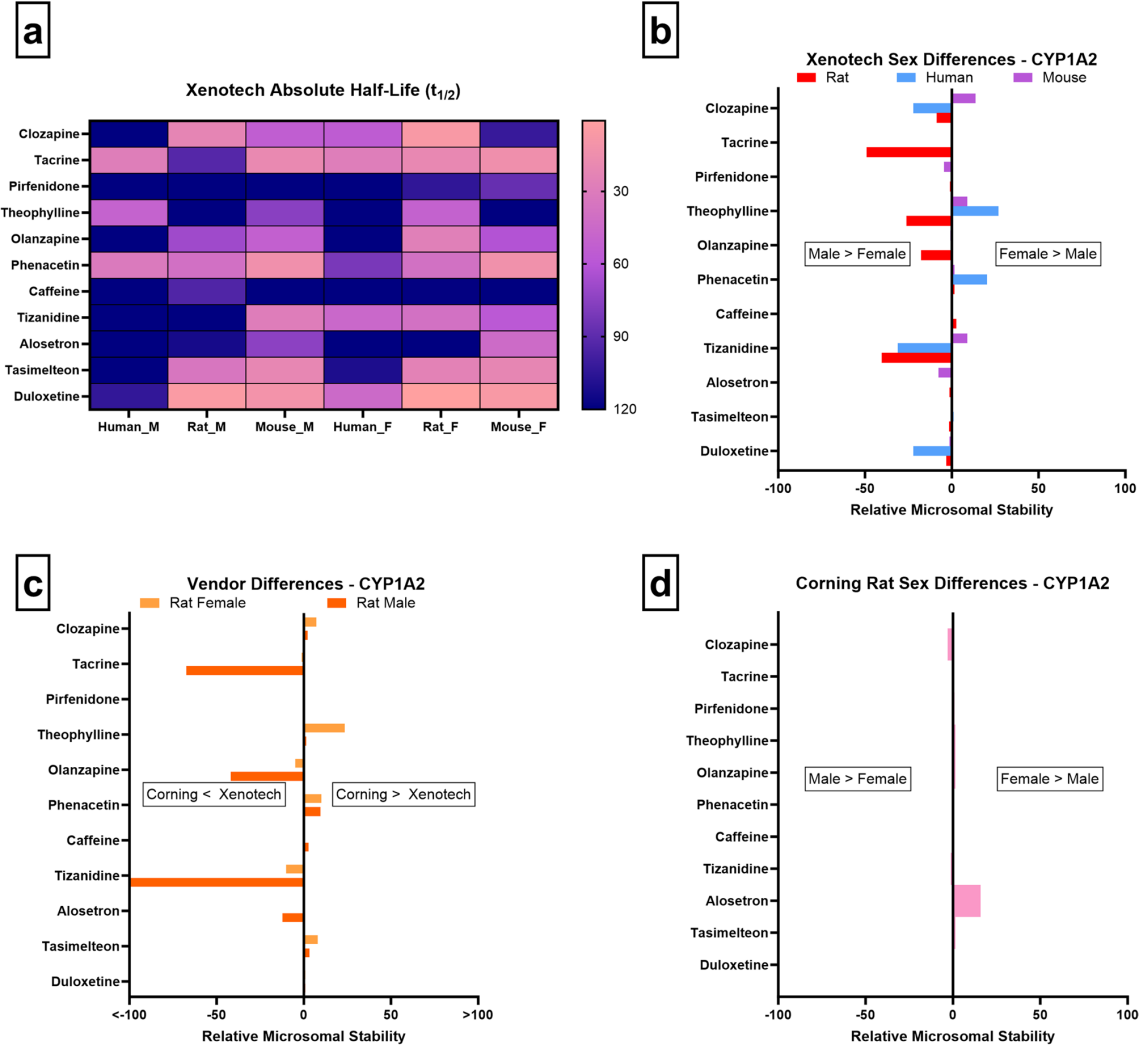
CYP1A2 microsomal stability comparisons across sex, vendor, species, and absolute half-life values. (**a**–**d** as above)

As shown in Fig. 7a/b, tacrine, theophylline, olanzapine, and tizanidine are more stable in male rat microsomes, indicative of lower rat Cyp1a activity in males and a female-predominant profile in this species. Human microsomes showed substrate specific and in some cases, such as clozapine, tizanidine, and duloxetine, demonstrate greater stability in males, suggesting higher CYP1A2 activity in females. However, theophylline and phenacetin display the opposite pattern, underscoring the substrate-dependent nature of CYP1A2 metabolism in humans. No consistent sex-driven differences are seen in mouse data from either Xenotech (Fig. 7a/b) or Corning.

Vendor comparisons in rats reveal significant variability; tacrine, olanzapine, and tizanidine show higher stability in Xenotech male rats relative to Corning males, while Xenotech males also display greater stability than their female counterparts (Fig. 7c). These trends were not reproduced in Corning male vs. female comparisons, suggesting that inter-vendor differences, rather than sex, may drive these discrepancies in certain cases (Fig. 7d).

Collectively, our findings are consistent with the broader literature in that CYP1A2-related activity is sex-influenced in both rats and humans but is also substrate-specific. This is further confounded by environmental factors such as induction^2^. The vendor-dependent discrepancies observed in rats from this study highlight the necessity of considering both supplier and sex effects in early-stage metabolic evaluations to avoid misinterpretation of CYP1A2-mediated clearance.

## Summary and conclusion

This study consolidates evidence of sex- and species-dependent variability in CYP450-mediated metabolism across humans, rats, and mice, while incorporating vendor-specific comparisons to highlight additional sources of experimental variation. Our results demonstrated distinct patterns in microsomal stability by sex and species for selected CYPs, illustrating the translational limitations of rodent models for predicting human PK. For instance, rodents exhibit a higher liver-to-body weight ratio (~ 4–5% vs. ~2.5% in humans), greater hepatic blood flow, and faster intrinsic microsomal clearance^5,57^. These factors collectively accelerate metabolic turnover compared to humans, contributing to rodents’ shorter drug half-lives and lower in vitro stability. Enzymatically, mice and rats express unique Cyp2c isoforms that lack direct human counterparts. Humans, by contrast, rely on single dominant isoforms (e.g., CYP2C9, CYP2D6, CYP3A4), where genetic polymorphisms and hormonal modulation exert greater influence than GH^2,57^. These regulatory and isoform-level differences explain why rodent models often exaggerate sex effects and clearance rates relative to human observations^7,27,57^. The review article by Martignoni et al., 2006 is an excellent resource for understanding key physiological and enzymatic differences amongst different species in context of drug metabolism^5^.

Together, these physiological and enzymatic contrasts emphasize why sex-related findings in rodents cannot always be directly extrapolated to humans. Accounting for sex differences is not only biologically important but also clinically necessary. Women are 1.5–1.7 times more likely to experience adverse drug reactions than men^7,55^. Sex differences in CYP activity affect drug efficacy and toxicity, as seen in examples like faster acetaminophen clearance in men^3,7^ or sex-dependent opioid response linked to CYP2D6 phenotype^7,59^. The NIH now recommends incorporating sex as a biological variable in experimental design^7,59^. Ignoring these differences during early-phase screening and preclinical testing can lead to costly failures in later stages of development due to unexpected efficacy or toxicity issues in one sex. For instance, in 2013 the Food and Drug Administration (FDA) showed data that women are at higher risk for excessive daytime sedation and impaired driving after bedtime doses of zolpidem. After being released into the market, the FDA reduced the recommended dosage for women in half^64^.

 In vitro observations of sex-based differences in CYP activity align with clinical findings for specific substrates and conditions, reinforcing the translational relevance of these models, while also highlighting cases where species or vendor effects can distort the signal. The substrates tested in this study, including cyclosporine^4,55^, ifosfamide^3^, ondansetron^2^, desipramine^2,4^, nortriptyline^4^, propranolol^2^, diazepam^4,55^, piroxicam^2^, caffeine^2,4,54^, olanzapine^2,4^, and clozapine^2,4^ have documented sex differences in patient populations. For instance, women exhibit faster clearance of cyclosporine and diazepam, more rapid N-dechloroethylation of ifosfamide, and slower clearance of ondansetron, nortriptyline, olanzapine, and clozapine, while men show higher clearance for substrates such as propranolol, caffeine, and piroxicam. While differences exist, it may not necessarily be clinically relevant for every compound but recognizing them upfront remains important for anticipating toxicological liabilities, informing first-in-human dosing, and improving PK/PD predictions.

The case of E2027, with its ~ 11-fold AUC difference between high- and low-clearance Sprague–Dawley rats, alongside the in-house compound showing both vendor-related microsomal variability and a 12-fold sex-based AUC disparity, underscores how rat-specific CYP polymorphisms, sex-dependent metabolism, and vendor variability can undermine PK predictions. Male-only microsomal screening in the latter failed to detect these liabilities, resulting in mismatches between in vitro and in vivo data, prompting repeat PK studies. Sex- and vendor-related differences can also confound toxicity assays such as the Ames test, potentially skewing mutagenicity outcomes. The Ames test is a rapid screening tool for identifying compounds with mutation-causing potential, widely used in drug discovery, chemical safety assessment, and environmental monitoring. It employs specific strains of Salmonella typhimurium or Escherichia coli engineered to be histidine- (or tryptophan-) deficient, which are exposed to the test compound with and without metabolic activation using a rat liver S9 fraction. Mutagenic compounds trigger genetic changes that restore the bacteria’s ability to produce histidine, resulting in colony growth on histidine-free plates. When sex- or vendor-driven biological variability enters the equation, it can distort these results, leading to false conclusions, wasted resources, and costly setbacks in the drug discovery pipeline.

These findings highlight three critical risks in preclinical workflows:

1: Species and colony variability - Unrecognized CYP polymorphisms in common lab rat strains can produce misleading PK profiles, potentially misguiding candidate selection.

2: Sex-Based Differences - Ignoring female metabolism may overlook liabilities or opportunities for dose optimization, delaying development and risking suboptimal trial design.

3: Vendor Effects - Variability in microsomal preparations can alter stability readouts, affecting clearance predictions as well as toxicity assessments (e.g. Ames tests, micronucleus tests).

Addressing sex, vendor, and genetic variability early in in vitro models can substantially improve translational accuracy, reduce attrition, and save time and resources in the already high-cost drug development process. By integrating comparisons across sex, species, and vendors, this study advocates for a multidimensional approach to early PK evaluation. While rodent and microsomal models cannot fully replicate human drug disposition, they remain indispensable for providing mechanistic insights and directional guidance. Proactively incorporating these factors enhances their predictive value, better aligning in vitro and in vivo findings with human PK and ultimately enabling safer, more effective drug development.

## Data Availability

All data generated or analyzed during this study are included in this published article.
